# Melanins in Fossil Animals: Is It Possible to Infer Life History Traits from the Coloration of Extinct Species?

**DOI:** 10.3390/ijms19020230

**Published:** 2018-01-23

**Authors:** Juan J. Negro, Clive Finlayson, Ismael Galván

**Affiliations:** 1 Department of Evolutionary Ecology, Doñana Biological Station—CSIC, 41092 Sevilla, Spain; galvan@ebd.csic.es; 2 The Gibraltar Museum, Gibraltar GX11 1AA, UK; clive.finlayson@gibmuseum.gi; 3 Department of Anthropology, University of Toronto, Scarborough, ON M1C 1A4, Canada

**Keywords:** dinosaur, fossil, paleo-color, skin coloration, countershading, pigments, melanin, melanosome

## Abstract

Paleo-colour scientists have recently made the transition from describing melanin-based colouration in fossil specimens to inferring life-history traits of the species involved. Two such cases correspond to counter-shaded dinosaurs: dark-coloured due to melanins dorsally, and light-coloured ventrally. We believe that colour reconstruction of fossils based on the shape of preserved microstructures—the majority of paleo-colour studies involve melanin granules—is not without risks. In addition, animals with contrasting dorso-ventral colouration may be under different selection pressures beyond the need for camouflage, including, for instance, visual communication or ultraviolet (UV) protection. Melanin production is costly, and animals may invest less in areas of the integument where pigments are less needed. In addition, melanocytes exposed to UV radiation produce more melanin than unexposed melanocytes. Pigment economization may thus explain the colour pattern of some counter-shaded animals, including extinct species. Even in well-studied extant species, their diversity of hues and patterns is far from being understood; inferring colours and their functions in species only known from one or few specimens from the fossil record should be exerted with special prudence.

## 1. Introduction

Color reconstruction in fossil species is an exciting scientific topic adding a realistic and more vivid touch to our perception of life on Earth in a distant past. Examples abound in the scientific literature [1,2,3,4] and this is already affecting the way paleo-artists and filmmakers are depicting dinosaurs and other long-vanished species. They are now increasingly pictured as brightly colored creatures, very much as many extant reptiles or birds look nowadays. The past is indeed very deep, and paleo-color investigations range widely in time and also in the taxa involved, covering both invertebrates and vertebrates. For instance, color reconstructions have been attempted for a 10-million-year-old (Ma) snake [5], 47 Ma moths [6], and a 99 Ma amber-preserved feathered dinosaur [7]. The color-generating mechanisms also vary, including different pigments such as melanins and carotenoids, or structural colors (e.g., [6]).

However, color determination in fossils is not necessarily an end in itself, and some investigations have sought functionalities for colors and patterns, be it sexual attraction [8] or plain crypsis. Two recent papers [9,10] have reported the color reconstruction of one individual dinosaur each (*Psittacosaurus* sp. and *Borealopelta markmitchelli*, respectively), both of which were seemingly countershaded for camouflage, with a darker dorsal coloration and a lighter ventral area. Vinther et al. [9] suggested that countershading in *Psittacosaurus* would be more protective under vegetation cover and concluded that it inhabited forests with a dense canopy. Brown et al. [10], in turn, remarked the fact that *Borealopelta* was much larger than any extant species showing countershading, and that it must have been preyed upon by gigantic predatorial dinosaurs. This would be proof that predator–prey dynamics in the Jurassic are different compared to today.

The color patterns of *Psittacosaurus* were analysed using two approaches. First was the observation, noted in a previous study [11], that the integument varied in the presence of dark-colored patches alternating with lighter ones. Vinther et al. [9] interpreted the dark materials as residues of melanins from original skin pigmentation. Second, the authors discovered in these darker areas some granular structures consistent with granules composed of pheomelanin. The same interpretation has been made previously by other fossil investigators [8], but not without controversy [12]. Brown et al. [10] used several analytical techniques that rendered significant amounts of benzothiazole, which according to them would be diagnostic for the presence of pheomelanin. Following this interpretation, the general color of *Borealopelta* would be reddish-brown on the back and whitish on the belly. A review of the literature and an assessment of the evidence in support of such interpretations, particularly concerning mechanisms responsible for melanin production, are therefore opportune.

The colors of animals are generally produced by deposition in the integument of several chemical forms of pigments, mostly melanins, which are synthesized by animals, and carotenoids, which are synthesized by plants and necessarily incorporated by animals from the diet [13]. Specialized integumentary structures also produce structural colors, often in combination with melanins, as with iridescent coloration [14], but also in the absence of these pigments [15]. 

We take for granted that dinosaurs and other extinct taxa were colored by melanins, as these are the pigments that typically give coloration to animals. However, precise integumentary color reconstruction, including patterns and actual color shades, is difficult based on what is left in fossils. In the same way as DNA, now known to irreversibly degrade with time, with previous claims of 80-million-year-old sequences proven to be artefactual [16,17], care must be exerted in color inferences based on fossilised structures. Again, our point is not to cast doubts on the presence of melanins in vanished species, but knowing that just two melanin types (eu- and pheomelanin) in combination with white unpigmented hair or skin provide the highly diverse coat color spectrum of Class Mammalia (see [13] for a rare exception), it is hard to explain fossil coloration. It is even more problematic to assign precise functionalities or, from there, infer the ecological niche, habitat preferences, or predator–prey relationships, of the species involved. 

The coloration of any animal may be the result of natural selection and/or sexual selection. Many authors have remarked that both types of processes may operate together: natural selection typically concealing animals in their environment, and sexual selection acting in the opposite way, making animals more conspicuous (at least at close range) [18]. However, this is an oversimplification, and animal colors may have evolved by natural selection for a number of reasons, including physical protection in the environment, concealment, advertising, and deception. Each of these broad categories may, in turn, be sub-divided into different operating mechanisms (Table 1). Countershading is just one of many possible factors by which the coloration of an animal may be adaptive.

## 2. Mechanistic Considerations

Fossils yielding carotenoid-based and/or structural coloration are extremely rare [5,6]; thus the majority of studies on the colors of extinct animals concern melanins. On the basis of the discovery of fossil melanin granules in the integument of a diversity of ancient vertebrates, it has been claimed that such information represents a window to the color patterning of extinct animals, or, more excitingly, a venue to the unravelling of aspects of the life history of these animals [20]. In our opinion, claims based on the analysis of fossilized melanin granules are not yet fully justified and should be reconsidered. 

Our arguments are as follows:

1. Melanins are the most ancient pigments in living organisms and the most widespread in animals. They appear from bacteria to humans in virtually all extant organisms [21]. For instance, except for albino mutants, all birds seem capable of synthesizing melanins [22]. The possibility exists that pigments different from those present in extant species evolved and disappeared in the past. If, as an example, all extant parrots (Order Psittaciformes) and turacos (Order Musophagiformes) vanished for any reason, the pigments that have evolved only in these birds, i.e., psittacofulvins and turacins [23], would vanish forever along with these lineages. This means that the complete disappearance of pigment classes should be accompanied by extinctions of entire lineages, which is highly improbable unless the given lineages are small and phylogenetically restricted. If this was the case, the pigments would not be very representative of the coloration of the groups: for example, the exceptionally vivid colors displayed by parrots and turacos do not represent the color patterns commonly observed among all extant birds.

However, no study to date has provided any evidence of pigment extinctions. Therefore, dinosaurs’ colors—and the pigments involved—as with other extinct animals, cannot be expected to have been really unique or special: they were probably much like the colors of extant animals, as they had at their disposal the same raw materials for pigmentation. The presence of melanins in dinosaurs would be as unsurprising as four limbs and two eyes.

2. Although extinct animals must have displayed conventional colors produced by melanins, an open question that still should be addressed is the exact color hues and the patterns they created. This is because, as it has recently been claimed [9,10], color patterning may provide clues on the ecology of animals. We have, however, two reservations about such claims. The first is that deciphering the ecology of extinct animals through their color is still out of reach. The second concern is that fossilized melanin granules provide, at best, information on the presence of the two main chemical forms of melanins (i.e., eumelanin and pheomelanin; e.g., [12]), but this is notenough to determine the exact color patterning of extinct species. A long-standing assumption is that eumelanin is a dark polymer that gives rise to colors from black to grey, while pheomelanin produces lighter, rufous colorations, and this has been embraced by most studies on preserved melanin granules to make claims about our capacity to infer the color of extinct animals [1,4]. This is, however, insufficient to infer color patterns with enough detail, and in the best of cases it may only serve to determine whether animals displayed contrasted patterns of dark and light color patches. Thus, fossilized melanin granules can be used to determine relative dark and light body patterns (in the case that the chemical nature of melanins can be obtained; see below), but not the actual colors exhibited by these body regions.

It must be noted that the chemical heterogeneity of melanins is not limited to just eumelanin and pheomelanin. Eumelanin is composed of 5,6-dihydroxyindole (DHI) and 5,6-dihydroxyindole-2-carboxylic acid (DHICA) moieties, both also forming the corresponding orthoquinones, while pheomelanin is composed of benzothiazole and benzothiazine moieties [24]. These different molecules render different colors in the animal integument, but the exact correspondence between the detailed melanin composition and the colors produced has not been determined until recently, when it was established that it is only the concentration of DHICA and benzothiazole moieties that affects the perceived variation in color across species [25]. Thus, unless a detailed chemical composition of melanins from fossils was obtained, the exact conformation of color patterns in extinct animals will not be known.

Chemical degradation analyses that offer information on DHI and DHICA content in fossil eumelanin granules have been attempted [26,27], but it is still unclear whether similar analyses will be possible for fossil pheomelanin. In any case, the precise determination of color patterning in extinct animals depends on the inference of the chemical composition of preserved fossil eumelanin and pheomelanin granules, something that has not been accomplished yet. We therefore recommend that future studies on the color of extinct animals include detailed analyses of the chemical composition of fossilized melanin granules beyond the eumelanin-pheomelanin level, as this is the only way to determine the exact colors generated by melanins [25]. It is uncertain whether it will be possible to apply degradative analyses of melanins, which presently represent the most reliable method for identifying melanin subunits, to fossilized melanin granules, but in recent years Raman spectroscopy has arisen as a powerful tool for the non-invasive analysis of melanins in the tissues of different organisms [28,29,30], and has even been applied to the fossilized melanins of an extinct bird [31]. The identification of melanins beyond the eumelanin-pheomelanin level is not yet possible with Raman spectroscopy, but the fact that this field is rapidly growing makes us believe that paleontologists interested in the color of extinct animals should consider increasing the use of this technique in the near future.

3. An additional concern is that, assuming that differentiating eumelanin and pheomelanin granules in the fossil record was enough to infer color patterns (which is not the case, as explained above), such differentiation should be made properly and consistently. However, eumelanin and pheomelanin identification has largely been made on the basis of the morphology of fossil melanin granules. The term melanosome is frequently used in paleo-color studies but not correctly, as it actually refers to a functional organelle in a melanocyte and not to melanin bodies that are pumped out of the organelles to be deposited in integumentary structures [21]. The morphology of melanin granules is not a reliable indicator of melanin composition. Indeed, several studies have based their conclusions on the assumption that eumelanin granules are rod-shaped structures while pheomelanin granules are spherical, which has thus constituted the basis of a large number of inferences made in the field of paleo-color [1,2,8,32,33]. Such an idea is based on previous studies [34,35]. However, Zi et al. [34] did not provide any chemical evidence that the rod-like granules that they found in the barbules of peacock *Pavo cristatus* feathers were actually composed of eumelanin, and Liu et al. [35] used a rather aggressive procedure to extract melanins from a hair matrix. As pheomelanin granules are less resistant to mechanical stress than eumelanin granules, such treatments might break pheomelanin granules making them appear spherical in contrast to unaltered eumelanin granules [18]. In fact, Liu et al. [35] already noted that both spherical and rod-shaped granules were observed in pheomelanin-colored hairs. This contradicts the idea that pheomelanin granules are spherical, an assumption later embraced by paleo-color studies.

Colleary et al. [12] employed a less aggressive extraction procedure and found rod-shaped granules in red chicken feathers, which are known to contain large amounts of pheomelanin [36]. This observation also contradicts the assumption that pheomelanin granules are spherical. Another study [37] using synthetic melanins found similar heterogeneity in the shape of eumelanin granules. Furthermore, it is known that the morphology of melanin granules change with age in humans [38,39]. Additionally, Liu et al. [35] only investigated melanin granules from human hair, and this fact alone demands further caution in extrapolating the results to other species in studies on fossil animals. To summarize, morphology does not seem to be a valid criterion to differentiate between eumelanin and pheomelanin in fossil melanin granules, still less to infer skin color in extinct animals. Furthermore, the fact that pheomelanin is less resistant to mechanical stress than eumelanin makes it likely that pheomelanin granules exhibit a higher speed of degradation than eumelanin granules. If some parts of the bodies of animals differentially degrade, this means that eumelanin and pheomelanin granules do not have the same likelihood of being in different parts of fossilized animals, which to our knowledge has never been considered.

Not all studies on the color of extinct animals base their conclusions on melanin composition inferred from the morphology of melanin granules, however. Some researchers [1,3,8,40] have averaged the morphology and density of melanin granules in modern feathers and hairs, and then associated these characteristics to the color of feathers or hairs as a whole. This approach saves the limitations of the assumptions on granule morphology and melanin composition, but in contrast ignores the association between morphology and color for individual melanin granules, leaving uncertain how the perceived color of feathers can be determined when only the morphology of a small fraction of their melanin granules is available. It must be noted that the color of melanins is given by their absorbance properties and not by the morphology of the granules that these polymers form [25]. Thus, advancement of the science of paleo-color will depend more on precise analyses of the chemistry of fossil melanin granules than on their morphological studies.

## 3. Functional Considerations

Let us assume that the color assignments in [9,10] were correct: we do have contrasting colored specimens in the range of hues provided by melanins: from black or reddish-brown to light yellowish, as numerous extant birds and mammals are colored today [25]. What we also know from extant terrestrial vertebrates is that sexual dichromatism is widespread [41], and that there are also numerous species showing color differences related to age. There are even bird and mammal species inhabiting high mountain areas and most northerly latitudes in the Northern Hemisphere that fully change fur or plumage seasonally from melanic brown to white, tracking winter snow cover. This includes nine extant mammal species, such as the arctic fox (*Alopex lagopus*) and the variable hare (*Lepus timidus*) [42], or birds such as the ptarmigan (*Lagopus muta*) [18]. 

**Figure 1:**
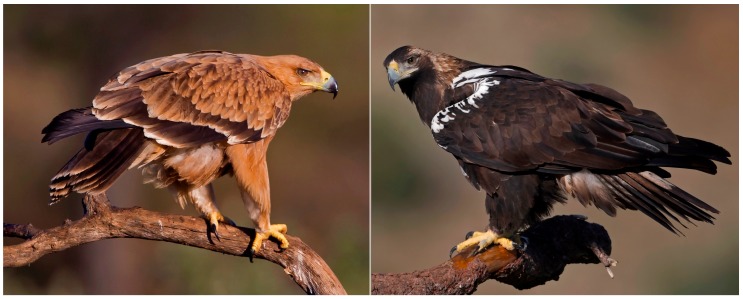
Spanish Imperial Eagles *Aquila adalberti*. On the left, a juvenile individual showing a light-colored plumage mainly based on the presence of pheomelanin. On the right, an individual showing the much darker full adult plumage (with white “shoulders”), based on eumelanin. This eagle species presents delayed plumage maturation, and the adult plumage is typically attained when individuals are 4 years old. Photographs by Stewart Finlayson, Gibraltar Museum.

The Spanish Imperial Eagle *Aquila adalberti* sports a full light-orange plumage coloration in juveniles whereas the adult birds are almost fully black (Figure 1). The same intraspecific variation can be found in mammals, for instance with the black-and-gold howler monkey *Alouatta caraya*. The adult male is pitch black, whereas adult females and juveniles are gold-colored. Were the single and only *Psittacosaurus* studied in [9], and the one *Borealopelta* specimen studied in [10], members of a monochromatic, dichromatic, or polychromatic species? With a sample size equal to one, this is impossible to assert. Curiously enough, *Psittacosaurus* is one of the few dinosaur genera for which a lifetime growth curve has been built based on fossil bone structures, and it has been proposed that they grew for several years before reaching the larger size [43]. For this curve to be generated, numerous specimens from the same paleontological site were used. Ideally, and following this example, paleoscientists should aim to describe integumentary coloration in a set of different individuals of different age classes and the two sexes, to determine whether the species under investigation showed intraspecific color variation.

There is of course the possibility that both *Psittacosaurus* and *Borealopelta* were fully invariant monochromatic species. But here comes the second contentious issue: considering this type of dual coloration as “countershading”. Also known as “Thayer’s Law”, it was stated as follows: “animals are painted by nature, darkest on those parts which tend to be most lighted by the sky’s light, and vice versa” [44]. This is indeed a widespread mode of coloration in animals, but the appropriateness of the term should be revised. The very word “countershading” implies a functional explanation: that the upper part of the body shades the lower part. This may be so in some animals with dual coloration, but not in all cases, and not in all situations, as it strongly depends on the animal’s orientation to the sun [45]. 

**Figure 2:**
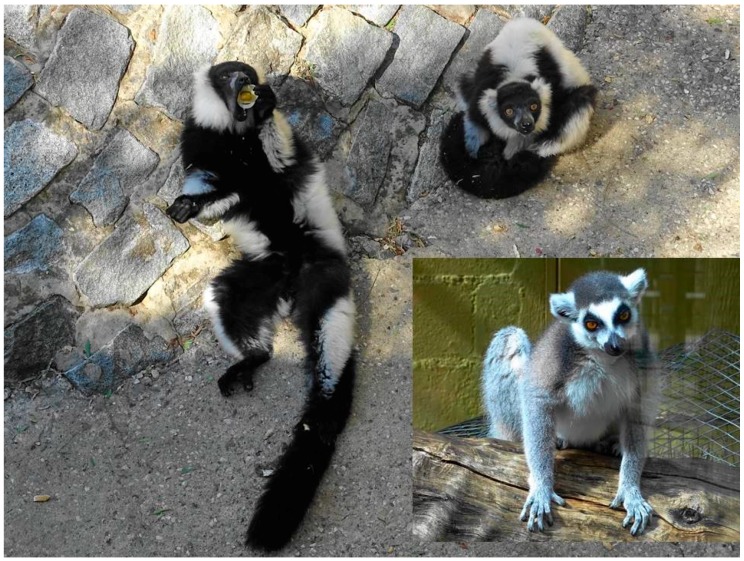
Two black-and-white ruffed lemurs *Varecia variegata*, one showing the black ventral area including the insides of the legs (**left**), and the other one showing the white dorsal area (**top**-**right**). These lemurs are arboreal, diurnal, and live in the rainforests of eastern Madagascar. In the lower right, a ring-tailed lemur *Lemur catta* showing a typical countershaded coloration, darker on top and white below. The ring-tailed lemur is the most terrestrial of all lemurs. It lives in forests and scrubland in southern Madagascar. Photographs taken at Zoobotánico Jerez (Spain) by Juan J. Negro.

Far from being a universal natural law, Thayer’s proposal has counter-explanations and there are also examples of “inverted countershading”—i.e., species in which the ventral area is darker than the dorsum, such as in the black-and-white ruffed lemur *Varecia variegata* (Figure 2), or countershaded species such as the bearded vulture *Gypaetus barbatus*, which darkens its white underparts purposely with iron oxides, constituting one of the most notorious examples of cosmetics in animals [46]. This is not, however, an isolated fact, as at least 28 bird species in 13 different families are known to alter their natural coloration cosmetically [47]. Non-biological adventitious pigments may thus be in the color palette of animals, including fossil species. 

The idea that countershading coloration is always a concealing adaptation has been contested by different authors [48,49] who pledged for the search of alternative explanations. The most parsimonious one is the economy of pigments [50,51]: whatever the functions of pigments, animals save in their production where they less need them. Eumelanin is ubiquitous among organisms because it fulfils a function that is key for life on Earth, i.e., protection against mutagenic UV radiation [52]. Melanins are polymers that in vertebrates are formed by the oxidation of the amino acid tyrosine in melanocytes, and thus relative metabolic costs will always be higher for melanocytes producing eumelanin than for inactive melanocytes [21]. Furthermore, melanin synthesis in melanocytes is stimulated by exposure to UV radiation [52] and thus unexposed melanocytes will always produce less pigment than exposed melanocytes, regardless of the costs of producing the pigment. Taken together, these two facts suggest that animals only deposit pigments in those body regions that are exposed to UV radiation because this saves metabolic costs and because melanocytes in the skin of these body regions are highly stimulated. The economy of pigments also applies to secondary functions fulfilled by melanins, such as visual communication [53], as shown by the fact that birds that use melanin-based plumage coloration for such functions only melanize those body regions that are displayed during the exhibitions with which they communicate with other birds [54].

**Figure 3:**
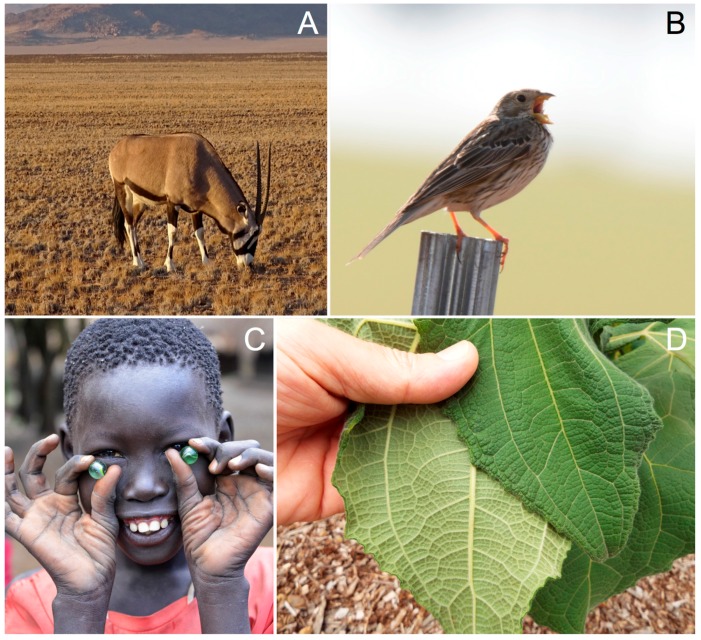
Different examples of contrasting coloration. (**A**) Gemsbok *Oryx gazella* from Namibia. Grey dorsally, white ventrally (Photograh by Juan J. Negro). (**B**) Corn bunting *Emberiza calandra*, a brown bird with a light belly (Photograph by Ismael Galván). (**C**) Dark-skinned person showing lighter coloration in the palms of the hands (credit and link: Rod Waddington; Available online: https://flic.kr/p/i3CAuX; covered by a CC BY-SA license (Available online, site last accessed 22/01/2018: https://creativecommons.org/licenses/by-sa/2.0/). (**D**) Yacón *Smallanthus sonchifolius* with a high color contrast between the upper side and underside of the leaves (credit and link: Forest and Kim Starr; https://flic.kr/p/DtS3hR; covered by a CC BY license (Available online, site last accessed 22/01/2018: https://creativecommons.org/licenses/by/2.0/).

The economy of pigments could be in fact the universal law sought after by Thayer and subsequent followers. Many mammals, and the majority of quadrupeds, place less pigment in ventral areas, including the inside of the legs. This is so both in wild species and in domesticated breeds. Could this always be for camouflage? We doubt it. Even humans have less pigment in the hand palms and feet soles—areas less exposed to the sun. The economy of melanin production cannot be more evident (Figure 3A–C).

Not surprisingly, integumentary pigments are sun-blockers in origin. Carotenoids, for instance, were “invented” by photosynthetic organisms to accompany chlorophyll for capturing excess photons [55] and some were later “recycled” by animals with the same essential function of photoprotection [56]. The economy rule of pigment allocation was in fact devised by green plants: the upper side and underside of leaves in many plants are different in color: darker dorsally and lighter ventrally (Figure 3D).

Last but not least, even assuming that *Psittacosaurus* and *Borealopelta* were monochromatic species and were indeed darker on top and lighter on the underside, can we still infer their behaviour and the general habitat they used to live in, or the size and ecology of their main predators? Optimal countershading may work best in environments with diffuse light, such as a forest understory [9], but how about a treeless green prairie or open savannah, such as the ones grazed by extant and countershaded species of gazelles, and with continuous or frequent overcasting? Light would indeed be diffuse during daytime in such a habitat. To make matters worse, a recent global analysis of bird plumage patterns in Class Aves revealed no association between habitat type and camouflage [57]. The relationship between the coloration of animals and the environments they live in is not always straightforward, except perhaps for white animals living in snow-covered areas [50]. Some relationships have been noted in the case of birds: desert species tend be melanized (dark), whereas oceanic birds that neither dive nor dig burrows tend to be white [58]. These relationships do not seem to respond to concealment needs but to entirely different factors, such as thermoregulation, abrasion-resistance, or social foraging (see Table 1 and [18]).

Pigment economization, compared to Thayer’s law, may look like a duller and simpler proposal. But what is the reason for so many contrastingly-colored animals in such a vast array of taxa and habitats around the world, now including even extinct species from other epochs? As with so many conundrums in Biology, a single solution may not work for all species. All explanations should be considered, including the less attractive ones, otherwise functional hypotheses derived from the presumptive color of extinct species may just be creative exercises of the imagination. 

Paleo-color is still a promising research field. Animal coloration does not only involve melanins and associated melanosomes; previous studies have, for instance, convincingly reported the finding of carotenoid-containing xantophores [5] or the presence of bright structural colors [6], perceived as blue or green. Paleoscientists should also be prepared to find fully or partially white (unpigmented) extinct species, and they should also keep in mind the numerous processes that may lead to adaptive colors and patterns, as detailed, for instance, in Table 1. Countershading is a possibility, but there is more beyond concealment, including intraspecific communication mediated by sexually-selected coloration. 

## Figures and Tables

**Table 1:** Mechanisms of color evolution by natural selection, adapted from [19] for animals at large, and Bortolotti [18], who specifically referred to birds. Four broad categories are recognized, although several mechanisms may combine to explain general coloration and specific markings in multi-colored species. Conspicuous colorations mainly resulting from sexual selection or some specific types of intraspecific communication (e.g., marks for species recognition, for gender and/or age recognition, or parent–offspring visual signaling) are not included.

**Protection**	Ultraviolet (UV)-protection. UV light is damaging to biological tissues. Abrasion-resistance. Sand and dust transported in the wind damages fur, feathers, and skin. Thermoregulation. Dark colors absorb more radiant energy than light colors.
**Concealment**	Crypsis. Blending in the environment by coloration and pattern. Countershading. Darker color on top compared to a lighter ventral area. Disruptive coloration. Irregular color patches to disrupt the shape of an animal in a variable environment.
**Advertising**	Unprofitable prey. Conspicuous colors in prey inform potential predators the prey is hard to get or distasteful. Allurement to prey. For instance, the red crest of kingbirds (*Tyrannus tyrannus*) attracting the bees they eat. Pursuit deterrence. Warning the presence of predators to conspecifics (e.g., white rumps or tails). Cohesion and coordination of the group. Black or white marks in flocking or bunching animal species, such as shorebirds, savannah gazelles, or fish schools. Startle or flash markings. Sudden appearance of conspicuous color patches in cryptic individuals to confuse predators and gain time to escape.
**Deception**	Resemblance to objects. This particular form of crypsis takes advantage of behavioural adaptations, such as slow motion in sloths, swaying movements in stick insects, and resting owls and frogmouths resembling tree bark or broken branches. False eyes (ocelli) or false faces. As in the wings of some butterflies or the nape of small owls and falcons. Directive marks. Intimidating markings, such as bright-colored irides, to scare off enemies or to obtain prey. Mimicry. Animals resembling others of a different species. The mimic is generally less poisonous or less powerful than the model.
